# The predictive value of systemic immune-inflammation index for vascular access survival in chronic hemodialysis patients

**DOI:** 10.3389/fimmu.2024.1382970

**Published:** 2024-05-17

**Authors:** Song Ren, Chuan Xv, Dongqing Wang, Yan Xiao, Panpan Yu, Deying Tang, Juan Yang, Xianglong Meng, Tao Zhang, Yaling Zhang, Qiang He, Quiang Li, Martin Gallagher, Yunlin Feng

**Affiliations:** ^1^ Department of Nephrology and Institute of Nephrology, Sichuan Provincial People’s Hospital, School of Medicine, University of Electronic Science and Technology of China, Sichuan Clinical Research Centre for Kidney Diseases, Chengdu, China; ^2^ Medical Information Center, Sichuan Provincial People’s Hospital, University of Electronic Science and Technology of China, Chengdu, China; ^3^ Nephropathy and Rheumatology, Medical Center Hospital of QiongLai City, Qionglai, China; ^4^ The George Institute for Global Health, University of New South Wales, Sydney, NSW, Australia

**Keywords:** hemodialysis, vascular access, survival analysis, systemic immune-inflammation index, prediction model

## Abstract

**Objective:**

To examine the prognostic values of systemic immune-inflammation indices of hemodialysis (HD) vascular access failure and develop a prediction model for vascular access failure based on the most pertinent systemic immune-inflammation index.

**Study design:**

A prospective cohort study.

**Setting & participants:**

Patients undergoing autogenous HD vascular access surgeries or arteriovenous graft as a permanent hemodialysis access in a tertiary center in southwest China from January 2020 to June 2022.

**Predictors:**

Systemic immune-inflammation indices, including NLR, dNLR, AAPR, SIRI, SII, PNI, PLR, and LIPI, and clinical variables.

**Outcomes:**

The outcome was defined as survival of the hemodialysis access, with both occluded and stenotic access being considered as instances of access failure.

**Analytical approach:**

Cox proportional hazard regression model.

**Results:**

2690 patients were included in the study population, of whom 658 experienced access failure during the follow-up period. The median duration of survival for HD vascular access was 18 months. The increased systemic immune-inflammation indices, including dNLR, NLR, SII, PNI, SIRI, PLR, and LIPI, are predictive of HD access failure, with SII demonstrating the strongest prognostic value. A simple SII-based prediction model for HD access failure was developed, achieving C-indexes of 0.6314 (95% CI: 0.6249 – 0.6589) and 0.6441 (95% CI: 0.6212 – 0.6670) for predicting 6- and 12-month access survival, respectively.

**Conclusions:**

Systemic immune-inflammation indices are significantly and negatively associated with HD vascular access survival. A simple SII-based prediction model was developed and anticipates further improvement through larger study cohort and validation from diverse centers.

## Introduction

The escalating prevalence of end stage renal disease (ESRD) is correlated with a growing population of patients reliant on dialysis, with hemodialysis (HD) being the predominant method (1). Success and durable vascular access is fundamental and essential for the success of regular hemodialysis treatment (2). Among the three main types of permanent vascular HD access, namely autogenous arteriovenous fistula (AVF), arteriovenous graft (AVG), and tunneled central venous catheter (CVC), AVF is associated with the lowest rates of complication (3) and is the preferred choice for permanent HD access whenever feasible.

Regrettably, vascular access failure frequently occurs. The failure of fistulas can be classified into two categories: maturation failure and secondary failure, with pre-existing vascular anomalies and venous lesions being the most probable causes, respectively. The most prevalent manifestations of secondary fistula failure are stenosis resulting from neointimal hyperplasia and occlusion caused by thrombosis (4). Emerging evidence suggested the vascular endothelium plays an important role in the remodeling and maturation of AVF (5, 6). The endothelium plays a crucial role in detecting hemodynamic changes following the creation of an AVF, and it also actively influences vascular remodeling by secreting vasodilatory factors and a series of proinflammatory molecules (7, 8). Additionally, the systemic microinflammatory status in ESRD also contributes to the development and exacerbation of endothelium dysfunction (5).

Various systemic immune-inflammation indices have been proposed to reflect the systemic inflammation status inside the body and have been proven to be effective prognostic indicators in a range of disease, particularly malignancies (9–13) and kidney disease (14, 15). Given the significant correlation between microinflammation status and neointimal hyperplasia, several studies have also examined systemic immune-inflammation indices in AVF (16–18). These studies have indicated the prognostic value of neutrophil-to-lymphocyte ratio (NLR) and systemic-immune-inflammation index (SII) for stenosis after AVG procedures (18), and platelet-to-lymphocyte ratio (PLR) for early AVF restenosis after percutaneous transluminal angioplasty (PTA) procedures (16). However, the existing research in this area remains limited and is characterized by small sample sizes and restricted types of procedure examined.

In order to investigate the outcomes of vascular access surgeries conducted in our hospital, a prospective cohort was initiated in January, 2020 and aimed to include all patients receiving permanent vascular access surgeries for hemodialysis in the Nephrology Department in Sichuan Provincial People’s Hospital. Since our department leads the diagnostic and therapeutic levels of vascular access dysfunction for hemodialysis in southeast China, the total annual volume of various HD access surgeries can reach up to approximately 1,000 cases, ensuring a substantial sample size of this cohort and comprehensive coverage of various scenarios for HD access failure. The laboratory results recorded in this cohort encompass all necessary components for the computation of systemic immune-inflammation indices, presenting a valuable opportunity to investigate the correlation between these indices and the outcomes of HD vascular access.

Therefore, the main objective of this study was to examine the prognostic values of eight systemic immune-inflammation indices that have been reported in literature, namely neutrophil-to-lymphocyte ratio (NLR), derived NLR, PLR, SII, albumin-to-alkaline phosphatase ratio (AAPR), lung immune prognostic index (LIPI), prognostic nutritional index (PNI), and systemic inflammation response index (SIRI), in relation to HD vascular access failure. The secondary purpose was to develop and validate a prediction model for vascular access failure based on the most pertinent systemic immune-inflammation index, in order to facilitate clinical practice.

## Methods

### Study design

This study is an ongoing, prospective, and observational cohort study conducted at Sichuan Provincial People’s Hospital encompassing all patients undergoing autogenous HD vascular access surgeries or AVG as a permanent hemodialysis access. The study commenced in January 2020 and does not involve any interventions. Eligible participants are adult patients (≥18 years old) admitted to our department who have been deemed by their treating physicians to require surgery related to a permanent hemodialysis access. These patients are invited to participate in the study prior to undergoing the index surgery.

The patients who provide consent to participate in the study are presented with an informed consent document that included a statement of a follow-up telephone interview to gather pertinent information regarding vascular access. Written informed consent is obtained from each patient upon their enrollment in the cohort. To ensure the evaluation of outcomes 12 months post-vascular surgeries for each patient, the population for this study was restricted to patients enrolled until June 2022. Patients who received tunneled CVC as their permanent access were not considered in this study. We also excluded patients who received autogenous vascular access surgeries on lower limbs.

This cohort study on hemodialysis vascular access is a component of a larger cohort study on chronic kidney disease which had been launched in our department since 2017. The cohort study was approved by the ethnic committee of Sichuan Provincial People’s Hospital (No. 2017.124). This study was exempted since this is an analysis of deidentified data. The study was conducted in accordance with the principles of Declaration of Helsinki for medical research involving human subjects. The report followed the TRIPOD (Transparent Reporting of a multivariable prediction model for Individual Prognosis Or Diagnosis) statement (19).

### Surgical procedures

All surgical procedures were conducted in Sichuan Provincial People’s hospital. The first-time arteriovenous fistula (AVF) and repair AVF surgeries were performed by one senior nephrologist in our department who had at least 5 years of experience in these specific access surgeries. The percutaneous transluminal angioplasty (PTA) surgeries were conducted under direct fluoroscopy by any of the three senior nephrologists (TZ, SR, and XLM) who possessed at least 5 years of experience in interventional peripheral vascular surgeries. Lastly, the prosthetic arteriovenous graft (AVG) implantation surgeries were carried out by either of the two senior authors (XLM and QH).

The type of surgical procedure was determined by the operating doctor. Each access surgery was counted as an independent surgery. The term “first-time AVF” denoted the initial creation of an AVF on either side of the upper limbs. Any subsequent surgery performed on the same side of the upper limb following the first AVF was counted as a repair AVF. The creation of a second AVF on the opposite side of the upper limb with the first AVF was classified as a first-time AVF.

### Follow-up schedule

The follow-up was conducted by a team of five specialized nurses in the Nephrology department who contact the patients via telephone interview every three months from the time of their surgeries until the occurrence of the outcome. The date of the index access surgery was considered as the starting time point. The nurses conducting the follow-up were not intentionally kept unaware of the specific procedures performed. During the follow-up, a simple and structured form was used to efficiently inquire about the status of the patient’s hemodialysis access, categorizing it as occluded, stenotic, or normal. If a patient presented to our department due to access failure prior to the scheduled follow-up time point, the resulting outcome was evaluated and documented during the corresponding admission. Subsequently, the follow-up was recommenced from the date of the repair surgery.

An occluded access was defined as an access lacking palpable tremor and confirmed to have no access blood flow by ultrasonography. A stenotic access was defined as an access with reduced blood flow and is incapable of sustaining a 4-hour dialysis session at a minimum blood flow rate of 150 ml/min. Both occluded access and stenotic access were considered as instances of access failure.

### Study outcome

The outcome was defined as survival of the hemodialysis access, with both occluded and stenotic access being considered as instances of no survival. The duration of survival was calculated from the date of the index surgery to the date of access failure, death or last contact.

### Collected variables

The database collected the following types of variables:

1. Clinical characteristics, including age and sex;
2. Types of access procedures;
3. Laboratory tests results obtained within five days prior to the surgery, including complete blood cell (CBC) counts, albumin (ALB), alkaline phosphate (ALP), lactate dehydrogenase (LDH), and C-reactive protein (CRP);
4. Access survival status and duration of survival (in months).

Eight systemic immune-inflammation indices in the existing literature were computed based on laboratory examination results prior to the HD access procedures, including neutrophil-to-lymphocyte ratio (NLR), derived neutrophil-to-lymphocyte ratio (dNLR), albumin-to-alkaline phosphatase ratio (AAPR), systemic inflammation response index (SIRI), systemic immune-inflammation index (SII), prognostic nutritional index (PNI), platelet-to-lymphocyte ratio (PLR), and lung immune prognostic index (LIPI) (12, 20–23) (see the details of calculation in **Supplementary Table 1**).

### Statistical analysis

Continuous variables were presented as mean ± standard deviation (SD) or median (interquartile range, IQR) based on normality testing results by Kolmogorov–Smirnov test. Categorical variables were reported as number (percentage). Statistical comparisons between groups were performed using Student’s t-test, Wilcoxon rank sum test, chi-square test, and Fisher’s exact test, as appropriate. Continuous variables were transformed into categorical variables based on the optimal cutoff values determined through receiver operating characteristic (ROC) curve analyses. Missing data were imputed using multiple imputation.

Survival analysis was carried out using the Kaplan–Meier curves and the log-rank test was employed to evaluate the differences in the access survival stratified by each systemic immune-inflammation indicator and baseline variable. For each systemic immune-inflammation indicator, a univariate Cox proportional hazard regression model was applied to estimate the hazard ratio (HR) and corresponding 95% confidential interval (CI), as well as the concordance index (C-index) and the corrected C-index after bootstrapping validation (n=1000).

The development population consisted of patients admitted from January 1, 2020 to December 31, 2021, while the temporal external validation population included patients admitted from January 1, 2022 to June 30, 2022. Candidate predictive variables were selected via a combination of (clinical) experience driven- and (statistical) data driven-approaches. Univariate Cox regression analyses of each systemic immune-inflammation indices and clinical variables were carried out. The systemic immune-inflammation index with the highest C-index was fitted into the forward stepwise multivariate Cox proportional hazard regression model, along with clinical variables with p values below 0.1 in the univariate Cox regression analyses, to develop a prediction model.

**Figure 1:**
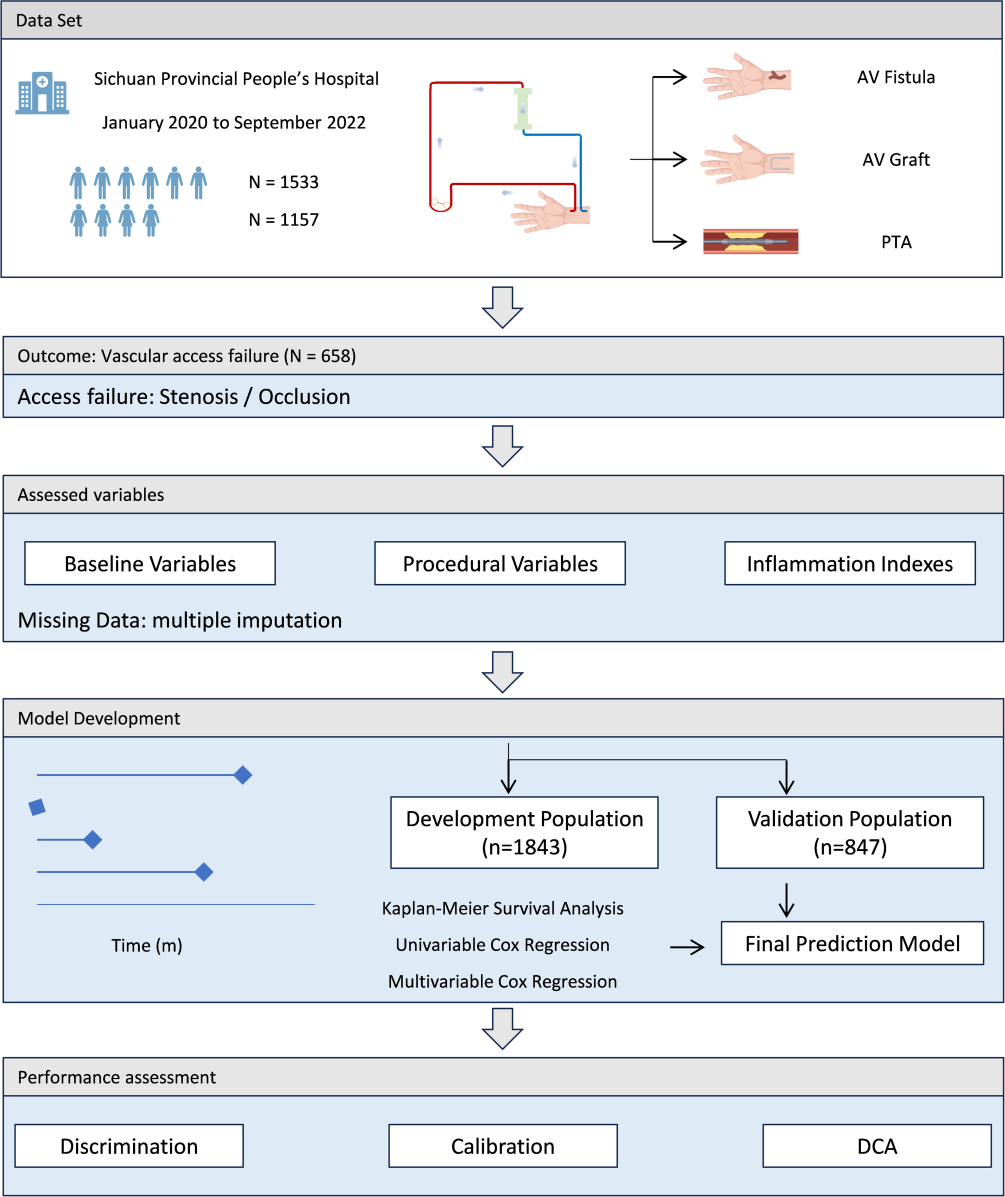
Schematic of the study workflow.

Performance assessment of the prediction model included three aspects. The discrimination performance was evaluated by the corrected C-index and corresponding 95% CI via bootstrapping (n=1000) internal validation, and the calibration performance was assessed by calibration plots for 6- and 12 months access survival. Decision curve analysis was performed to determine the net benefit of the prediction model. The performance in the external validation was evaluated using C-index and corresponding 95% CI, as well as calibration plots for 6- and 12 months access survival. The final model was converted into a nomogram to facilitate its applicability in clinical settings. The schematic of the study flow was shown in **Figure 1**.

All statistical analyses were conducted using R 4.0.3 (The R Core Team, R Foundation for Statistical Computing, Vienna, Austria) with the R packages “rms”, “survival”, “survivalROC”, “survminer”, and “dcurves” to access the performance of the prediction model, and “ggplot2” to plot the graphs. A two-sided p value less than 0.05 was considered statistically significant.

## Results

### Characteristics of the study population

A total of 3049 patients who had undergone autogenous AVF or AVG surgeries, either as a first-time or repeat procedure, were identified from the cohort database. Among this sample, 317 patients could not be reached for the initial follow-up telephone interview at three months after surgery and an additional 38 patients declined to participate in the interviews. Consequently, these individuals were excluded from the final analysis. Furthermore, four patients were excluded due to undergoing access operations on low limbs. As a result, 2690 patients were included in the study population, of whom 658 experienced access failure during the follow-up period. The median duration of survival for HD vascular access in the overall population was 18 (IQR: 11 - 28) months.

Patients who experienced vascular access failure exhibited several notable characteristics, including advanced age, a greater frequency of PTA procedures, and elevated levels of white blood cell count, lymphocyte count, neutrophil count, monocyte count, HGB, and CRP. Additionally, six out of the eight systemic immune-inflammation indices demonstrated significantly higher trends in patients with access failure. There was no significant difference in the distribution of sexes between the two groups. **Table 1** provides a comprehensive overview of the study population’s characteristics.

**Table 1:** Characteristics of the study population.

Variables	Total (n=2690)	Vascular access survival (n=2032)	Vascular access failure (n=658)	P value
Demographic				
Age, Median (IQR)	57 (48, 68)	57 (47, 67)	58 (49, 69)	0.021^*^
Sex, n (%)				0.113
Male	1533 (57)	1176 (58)	357 (54)	
Female	1157 (43)	856 (42)	301 (46)	
Procedural				
Procedures, n (%)				< 0.001^*^
First time AVF	1800 (67)	1396 (69)	404 (61)	
Repeat AVF	398 (15)	305 (15)	93 (14)	
PTA	391 (15)	258 (13)	133 (20)	
AV Graft	101 (4)	73 (4)	28 (4)	
Survival time (month), Median (IQR)	18 (11, 28)	21 (14, 30)	5 (0, 15)	< 0.001^*^
Clinical				
Lym, Median (IQR)	1 (0.75, 1.33)	1.02 (0.77, 1.34)	0.96 (0.69, 1.26)	< 0.001^*^
PLT, Median (IQR)	163 (120, 208)	157 (115, 201)	183 (136, 234)	< 0.001^*^
Neu, Median (IQR)	4.37 (3.38, 5.63)	4.19 (3.25, 5.31)	4.97 (3.82, 6.5)	< 0.001^*^
Mon, Median (IQR)	0.41 (0.3, 0.54)	0.4 (0.29, 0.52)	0.44 (0.33, 0.58)	< 0.001^*^
WBC, Median (IQR)	6.1 (4.97, 7.62)	5.94 (4.81, 7.34)	6.86 (5.44, 8.52)	< 0.001^*^
HGB, Median (IQR)	89 (75, 106)	88 (74, 105)	93 (78, 110)	< 0.001^*^
ALB, Median (IQR)	36.45 (31.3, 41.2)	36.3 (31.2, 41)	36.9 (31.8, 41.9)	0.057
ALP, Median (IQR)	85 (67, 113)	85 (66, 113)	89 (69, 113)	0.127
LDH, Median (IQR)	243 (195, 335)	243 (195, 330)	243 (196, 356)	0.414
CRP, Median (IQR)	3.43 (1.11, 10.28)	3.09 (1.04, 9.67)	4.75 (1.67, 16.54)	< 0.001^*^
Systemic immune-inflammation indices				
NLR, Median (IQR)	4.24 (3.09, 6.17)	4.01 (2.95, 5.59)	5.35 (3.67, 7.96)	< 0.001^*^
dNLR, Median (IQR)	2.56 (1.96, 3.46)	2.44 (1.89, 3.24)	3.05 (2.2, 4.15)	< 0.001^*^
AAPR, Median (IQR)	0.42 (0.3, 0.55)	0.42 (0.31, 0.55)	0.41 (0.3, 0.55)	< 0.001^*^
SIRI, Median (IQR)	1.72 (1.09, 2.81)	1.58 (1.03, 2.48)	2.28 (1.38, 3.91)	< 0.001^*^
SII, Median (IQR)	688.96 (434.8, 1074.47)	631.16 (403.39, 927.07)	977.57 (579.39, 1495.15)	0.517
PNI, Median (IQR)	41.62 (36.54, 46.74)	41.62 (36.53, 46.5)	41.63 (36.6, 47.23)	0.508
PLR, Median (IQR)	158.76 (115.56, 217.46)	150.67 (110.13, 202.03)	191.28 (134.83, 273.6)	< 0.001^*^
LIPI, n (%)				< 0.001^*^
Good	965 (66)	780 (71)	185 (53)	
Intermediate	1183 (45)	894 (45)	289 (45)	
Poor	489 (34)	322 (29)	167 (47)	
Survival status				
Occluded	305 (11)	0 (0)	305 (46)	< 0.001^*^
Stenostic	353 (13)	0 (0)	353 (54)	
Normal	2032 (75)	2032 (100)	0 (0)	

*Statistically significant.

AAPR, albumin-to-alkaline phosphatase ratio; ALB, albumin; ALP, alkaline phosphatase; AV, arteriovenous; AVF, arteriovenous fistula; CRP, C reactive protein; dNLR, derived neutrophil-to-lymphocyte ratio; HGB, hemoglobulin; IQR, inter quartile range; LDH, lactate dehydrogenase; LIPI, lung immune prognostic index; Lym, lymphocyte; Mon, monocyte; n, number; Neu, neutrophil; NLR, neutrophil-to-lymphocyte ratio; PLR, platelet-to-lymphocyte ratio; PLT, platelet; PNI, prognostic nutritional index; PTA, percutaneous transluminal angioplasty; SII, systemic immune-inflammation index; SIRI, systemic inflammation response index.

### Survival analysis of systemic inflammation indices

**Figure 2:**
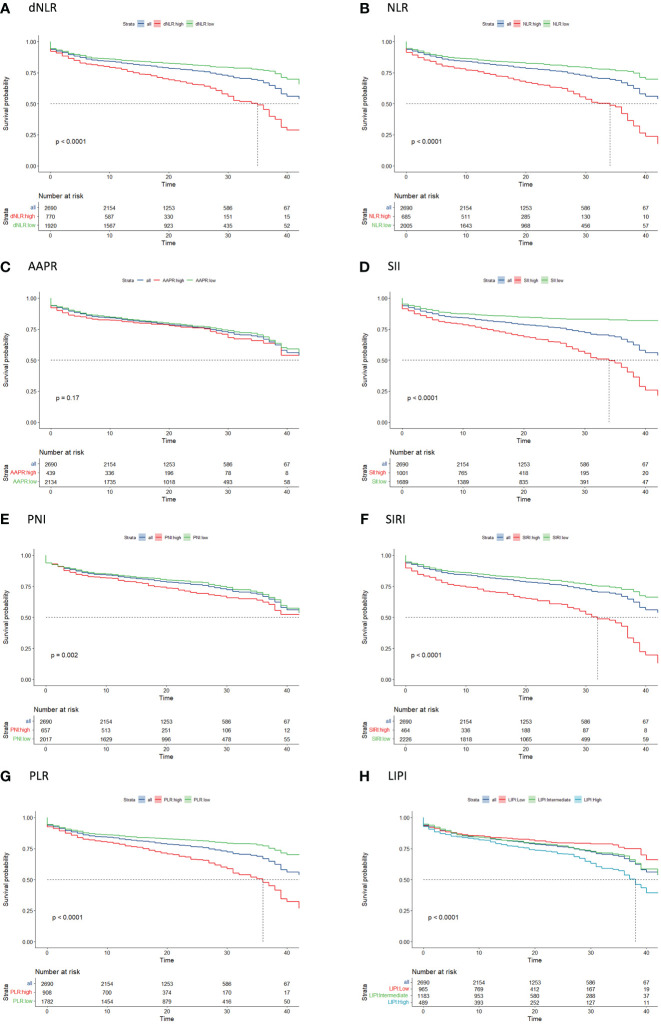
Kaplan-Meier curve analysis of access survival for each systemic immune-inflammation indicator including **(A)** dNLR, **(B)** NLR, **(C)** AAPR, **(D)** SII, **(E)** PNI, **(F)** SIRI, **(G)** PLR, **(H)** LIPI. AAPR, albumin-to-alkaline phosphatase ratio; dNLR, derived neutrophil-to-lymphocyte ratio; LIPI, lung immune prognostic index; NLR, neutrophil-to-lymphocyte ratio; PLR, platelet-to-lymphocyte ratio; SII, systemic immune-inflammation index; SIRI, systemic inflammation response index.

**Figure 3:**
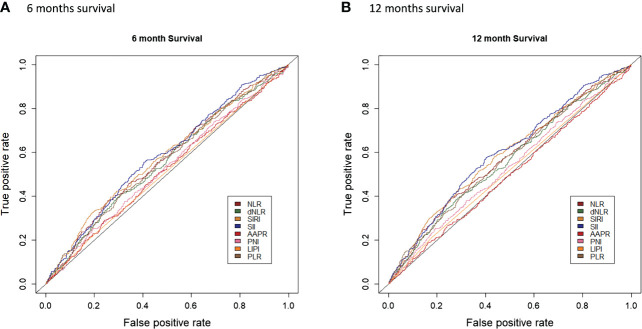
Comparison of the prognostic values of each examined systematic immune-inflammation indices for access failure at 6 **(A)** and 12 **(B)** months.

Univariate survival analysis for each systemic inflammation index revealed a significant association between HD access failure had high levels of dNLR (HR: 2.15, 95% CI: 1.84–2.51, p<0.001), NLR (HR: 2.37, 95% CI: 2.03–2.77, p<0.001), SII (HR: 2.77, 95% CI: 2.37–3.24, p<0.001), PNI (HR: 1.30, 95% CI: 1.10–1.54, p=0.003), SIRI (HR: 2.34, 95% CI: 1.98–2.76, p<0.001), PLR (HR: 2.08, 95% CI: 1.78–2.42, p<0.001), and LIPI (HR: 1.66, 95% CI: 1.34–2.04, p<0.001) (**Supplementary Figure 1**; **Figure 2**). The comparison of the prognostic values of each examined systematic immune-inflammation indices for access failure at 6 and 12 months showed the SII exhibited the highest C-index compared to the other indices examined. As a result, the SII was selected as the appropriate systemic immune-inflammation indicator to be fit into the Cox regression model (**Figure 3**; **Supplementary Table 2**).

### Model development and performance assessment

The development population consisted of 1843 patients admitted between January, 2020 to December, 2021. The univariate Cox regression analyses of each candidate predictive variables using predetermined cutoff values indicated advanced age procedure type, elevated hemoglobulin level, and high ALB level were potential predictive factors of access failure with p values below 0.1. These four predictive variables, along with the SII, were then fitted into the stepwise multivariate Cox regression to develop the final model (**Table 2**). The final model included three significant predictive variables, i.e. procedure type (PTA: HR: 2.16, 95% CI: 1.67 – 2.80, p<0.001), hemoglobulin ≥ 98 g/L (HR: 1.40, 95% CI: 1.15 – 1.69, p<0.001), and SII ≥ 850.8 (HR: 2.82, 95% CI: 2.36 – 3.37, p<0.001) (**Table 2**).

**Table 2:** Univariate and multivariate Cox proportional hazard regression analysis of vascular access survival.

Predictive variables		Univariate regression			Multivariate regression		
		Coefficient	HR (95% CI)	p value	Coefficient	HR (95% CI)	p value
Age	Low (<67)	–					
	High (≥67)	0.165	1.18 (0.97 – 1.43)	0.093			
Sex	Male	–					
	Female	0.047	1.12 (0.94 – 1.33)	0.225			
Procedure type	First-time AVF	–			–		
	Repeat AVF	0.066	1.07 (0.83 – 1.38)	0.613	0.037	1.04 (0.80 – 1.35)	0.783
	PTA	0.845	2.33 (1.83 – 2.96)	<0.001^*^	0.770	2.16 (1.67 – 2.80)	<0.001^*^
	AV Graft	0.442	1.56 (1.04 – 2.32)	0.031^*^	0.378	1.46 (0.97 – 2.20)	0.069
HGB	Low (<98)	–			–		
	High (≥98)	0.680	1.66 (1.39 – 1.98)	<0.001^*^	0.333	1.40 (1.15 – 1.69)	<0.001^*^
ALB	Low (<41.1)	–					
	High (≥41.1)	0.326	1.39 (1.24 – 1.69)	0.001^*^			
SII	Low (<850.8)	–			–		
	High (≥850.8)	0.000	1.02 (1.01 – 1.03)	<0.001^*^	1.03611	2.82 (2.36 – 3.37)	<0.001^*^

*statistically significant.

ACR, albumin-to-creatinine ratio; BUN, blood urine nitrogen; CHF, chronic heart failure; CI, confidence interval; eGFR, estimated glomerular filtration rate; HGB, hemoglobulin; MAP, mean arterial pressure; SII, systemic immune-inflammation index.

The C-indexes of the final model for predicting 6- and 12-months survival were 0.6314 (95% CI: 0.6249 – 0.6589) and 0.6441 (95% CI: 0.6212 – 0.6670), respectively (**Supplementary Figure 2**). The calibration plots demonstrated superior calibration performance in the higher survival region (**Supplementary Figure 3**). The decision curve analysis revealed net benefits ranging from 10% to 25% and 10% to 30% for the prediction of 6- and 12- month access survival, respectively (**Supplementary Figure 4**).

### Performance in the external validation

**Figure 4:**
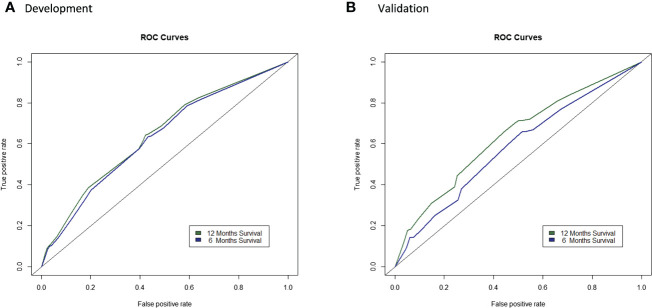
Comparison of ROC curves of the prediction model in the development and validation populations. **(A)** and validation **(B)** populations.

**Figure 5:**
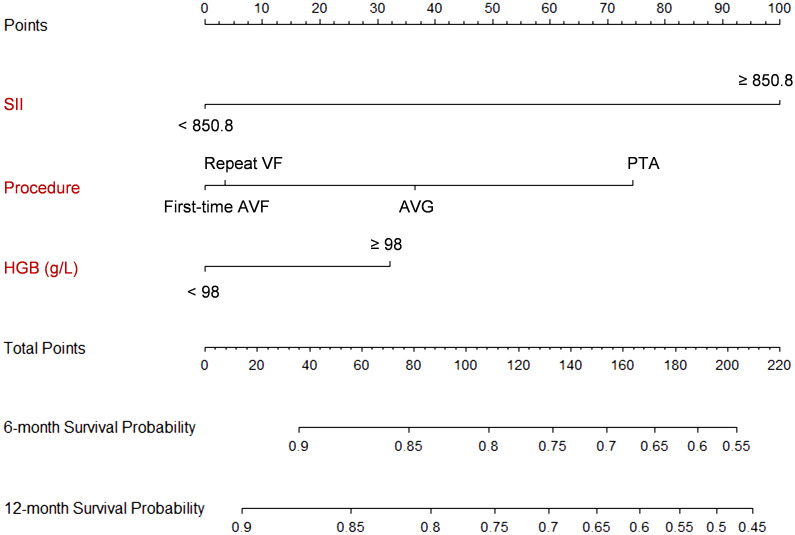
Nomogram of the prediction model. Each predictor is scored a corresponding point based on its value. The points are summed up to get a total point, which can be used to get the 6- and 12-month survival probability from the scales on the nomogram. For example, suppose a patients had a SII of 1000, receives a PTA procedure, and has a hemoglobulin level at 100 g/L. The total score is 100 + 72 + 30 = 202, which corresponds to a 6-month survival probability of 56% and a 12-month survival probability of 47%.

The C-indexes of the final model for predicting 6- and 12-months survival in the external validation were 0.5828 and 0.6347, respectively (**Supplementary Figure 5**). The calibration plots revealed relatively good calibration performance (**Supplementary Figure 6**). The decision curve analysis indicated better performance of the model in predicting 12-month survival compared to 6 months survival (**Supplementary Figure 7**). The comparison of ROC curves of the prediction model between the development and validation populations is presented in **Figure 4**. A nomogram was generated to facilitate clinical application (**Figure 5**).

## Discussion

The findings of this study show a significant association between HD vascular access survival and nearly all reported systemic immune-inflammation indices, including dNLR, NLR, SII, PNI, SIRI, PLR, and LIPI. The increased systemic immune-inflammation indices are predictive of HD access failure, with SII demonstrating the strongest prognostic value. A simple SII-based prediction model for HD access failure was developed, achieving C-indexes of 0.6314 (95% CI: 0.6249 – 0.6589) and 0.6441 (95% CI: 0.6212 – 0.6670) for predicting 6- and 12-month access survival, respectively. The model exhibited comparable performance for 12-month access survival in the development and external validation populations.

Our result of a significant association between elevated levels of systemic immune-inflammation indices with HD access failure aligns with previous studies (16–18). Furthermore, this study conducted a comparative analysis of various systemic immune-inflammation indices previously reported, and determined that SII exhibited the highest prognostic value. It is important to acknowledge that caution should be exercised when calculating the pre-operative SII, as the calculation equation may be influenced by potential acute-phase inflammation (23). Acute inflammation resulting from active infection or uncontrolled systemic inflammatory disease might unrealistically increase SII; however, its potential impact on the survival of HD vascular access survival remains uncertain. From this point of view, it may be advisable to delay the procedure until the acute inflammation has been effectively managed. Our observation that HD access failure, particularly failure of maturation, occurs at a higher rate among patients with concurrent infection or active inflammatory diseases such as ANCA vasculitis, further supports this approach. Endothelium dysfunction and edema, along with subsequent neointimal hyperplasia, may serve as potential underlying factors.

In comparison to patients who received first-time AVF procedures, patients who underwent PTA procedures exhibited a significantly higher risk of access failure, with a magnitude 1.16 times greater. Conversely, the risk of access failure in patients undergoing repeat AVF and AVG procedures was also elevated, albeit not statistically significant. The observed significant association between PTA and access failure might result from our practice, wherein AVF is prioritized as the preferred first choice, tunneled CVC is considered a last resort, and PTA is typically employed as a salvage treatment for vascular accesses displaying evidence of stenosis, rather than an early or regular intervention. The strategy of regular ultrasonography monitoring and timely PTA intervention (4, 24) has been recommended; nevertheless, its practical implementation may be hindered by constraints such as limited access to interventional radiology facilities, a scarcity of experienced interventional radiologists, and even insurance status of the patients (25). Another possible explanation could be that PTA addresses the stenosis, instead of providing a permanent resolution for the stenotic segment of veins, unlike repeat AVF or AVG procedures. This approach bears a remarkable risk of re-stenosis due to the limited use of stenting in this particular field (17). Furthermore, the observation that first-time AVF patients have the lowest risk of access failure implies that patients with access dysfunction should be more closely monitored and potentially require treatments to manage the risk factors for access failure, as its occurrence predicts an increased risk of reoccurrence in the future.

This study comprehensively examined the prognostic values of existing systemic immune-inflammation indices for HD vascular access in the largest cohort so far. It benefited from a well-organized cohort with close and regular follow-up, resulting in a relatively complete dataset. The lost-to-follow-up rate (355/3049, 11.6%) was low owing to a dedicated follow-up team comprising five specialist nurses. Routine preoperative tests such as CBC count, albumin, and ALP in our department further minimized the impact of missing baseline data on our findings, thereby facilitating the development and validation of our model. Additionally, our study holds the advantage of external validation through the utilization of a temporally distinct population. Despite of the differences between the development and external validation populations, the performance of the model for 12-month survival in the external population was comparable to that in the development population.

There are several noteworthy limitations that should be acknowledged. Firstly, it is important to recognize the substantial variations in the utilization and care of HD vascular access among geographically distinct HD centers. Since we receive patient from all over the southwestern region, a considerable proportion of patients return to their residencies following surgery where the functionality of vascular access may be influenced by disparities in the evaluation, care, and monitoring of vessels, all of which can impact the durability of HD access. The implementation of and strict adherence to standardized operating procedures for HD can be instrumental in elevating the quality of practice across diverse geographic regions. Second, the adjudicated outcomes obtained through telephone interviews may be subject to bias compared to the real situation, as the patients might be less well educated to precisely describe the state of their vascular accesses. In addition, since a large proportion of patients were not local residents and their results of outcomes came from follow-up interview, we can only assess the status of the access based on the feedback of the patients themselves. Therefore, the outcome assessment is functional in nature, not from accurate measurement of the peak systolic velocity diameter by vascular ultrasound examination. To mitigate the above potential bias, our strategy involves having the interviews conducted by senior specialist nurses who possess extensive training and years of experiences in HD vascular access. They would make every effort to communicate the interview questions in a clear and understandable manner, ensuring that patients are able to provide as much accurate information as possible. Third, the prioritization of different types of procedures varied across centers performing HD vascular access procedures. Given the significance of procedure type as a predictor in our model, the generalizability of this model necessitates validation from additional centers. Fourth, the C-index of our prediction model is mediocre. Expanding the study cohort with measures aimed at addressing the aforementioned limitations and seeking validations from centers located in diverse geographical regions might help to further improve the performance.

## Conclusions

In summary, this study showed nearly all reported systemic immune-inflammation indices are significantly and negatively associated with HD vascular access survival. A simple SII-based prediction model for HD access failure was developed which exhibited comparable performance in predicting 12-month access survival during external validation. Further improvement of the model is anticipated through larger study cohort and validation from diverse centers.

## Data availability statement

The original contributions presented in the study are included in the article/**Supplementary Material**. Further inquiries can be directed to the corresponding author.

## Ethics statement

The cohort study was approved by the ethnic committee of Sichuan Provincial People’s Hospital (No. 2017.124). Written informed consent is obtained from each patient at the entry of the cohort. This study was exempted for informed concent since this was an analysis of deidentified data.

## Author contributions

SR: Conceptualization, Data curation, Formal analysis, Methodology, Software, Validation, Writing – original draft, Writing – review & editing. CX: Data curation, Formal analysis, Investigation, Software, Writing – original draft. DW: Data curation, Formal analysis, Methodology, Writing – original draft. YX: Data curation, Formal analysis, Investigation, Validation, Writing – original draft. PY: Data curation, Formal analysis, Investigation, Writing – original draft. DT: Data curation, Formal analysis, Investigation, Writing – original draft. JY: Data curation, Investigation, Methodology, Writing – original draft. XM: Data curation, Methodology, Project administration, Writing – original draft. TZ: Data curation, Methodology, Resources, Writing – original draft. YZ: Data curation, Formal analysis, Methodology, Writing – original draft. QH: Conceptualization, Validation, Visualization, Writing – review & editing. QL: Methodology, Software, Validation, Writing – review & editing. MG: Methodology, Project administration, Resources, Validation, Writing – original draft, Writing – review & editing. YF: Conceptualization, Funding acquisition, Methodology, Software, Validation, Visualization, Writing – original draft, Writing – review & editing.
