# Supplementary material for: The predictive value of systemic immune-inflammation index for vascular access survival in chronic hemodialysis patients
Source: Front Immunol. 2024 May 17;15:1382970. doi: 10.3389/fimmu.2024.1382970 (PMC11140091; doi:10.3389/fimmu.2024.1382970)
Supplement: Supplementary file 1 [file Presentation_1.pptx]

## Slide 1
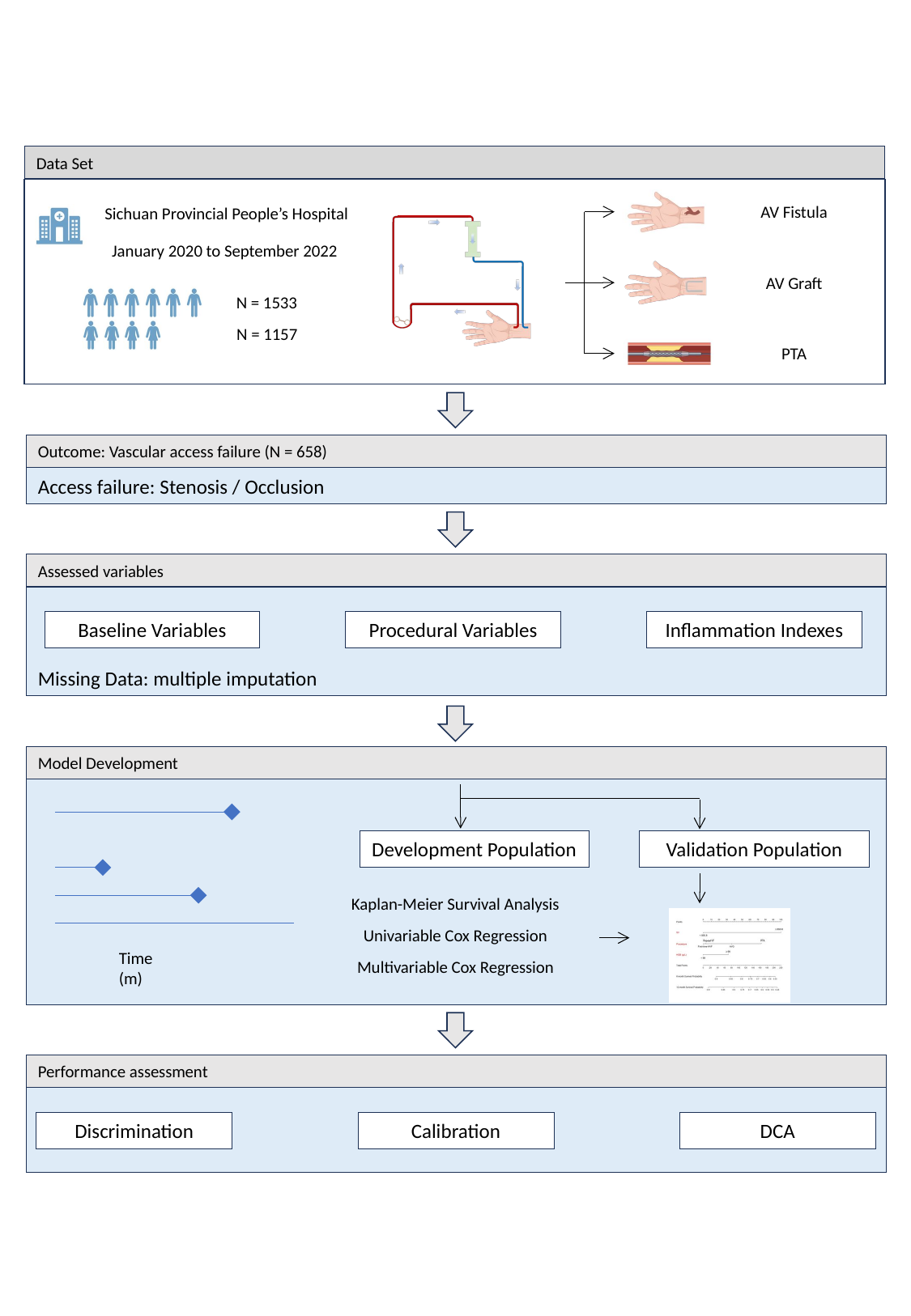

Data Set
AV Fistula
Sichuan Provincial People’s Hospital
January 2020 to September 2022
AV Graft
N = 1533
N = 1157
PTA
Outcome: Vascular access failure (N = 658)
Access failure: Stenosis / Occlusion
Assessed variables
Missing Data: multiple imputation
Baseline Variables
Procedural Variables
Inflammation Indexes
Model Development
Development Population
Validation Population
Kaplan-Meier Survival Analysis
Univariable Cox Regression
Time (m)
Multivariable Cox Regression
Performance assessment
Discrimination
Calibration
DCA
