# Supplementary material for: The predictive value of systemic immune-inflammation index for vascular access survival in chronic hemodialysis patients
Source: Front Immunol. 2024 May 17;15:1382970. doi: 10.3389/fimmu.2024.1382970 (PMC11140091; doi:10.3389/fimmu.2024.1382970)
Supplement: Supplementary file 2 [file DataSheet_1.docx]

**Supplementary Materials**

Supplementary Table 1. Calculation equations for the six systemic immuno-inflammation indexes.

Supplementary Table 2. Survival analysis for each systemic inflammation indexes.

Supplementary Table 3. Characteristics of the development and validation populations.

Supplementary Figure 1. Distribution of systemic immune-inflammation indexes based on cut-off values determined by ROC analysis.

Supplementary Figure 2. ROC analysis for the final model to predict 6- and 12- month access survival in the development population.

Supplementary Figure 3. Calibration plots for the final model to predict 6- and 12- month access survival in the development population.

Supplementary Figure 4. DCA curves for the final model to predict 6- and 12- month access survival in the development population.

Supplementary Figure 5. Discrimination assessment of the prediction model in the external validation population.

Supplementary Figure 6. Calibration assessment of the prediction model in the external validation population.

Supplementary Figure 7. DCA curves of the prediction model in the external validation population.

Supplementary Table 1. Calculation equations for the six systemic immuno-inflammation indexes.

| Indexes | Equations |
| --- | --- |
| NLR[1] | absolute neutrophil count (10^9^/L) / absolute lymphocyte count (10^9^/L) |
| dNLR[1] | absolute neutrophil count (10^9^/L) / (white blood cell count (10^9^/L)- absolute neutrophil count (10^9^/L)) |
| LIPI[2] | The LIPI was a categorized variable characterizing 3 groups:  1) Good: dNLR≤3 and LDH≤ ULN (250 U/L);  2) Poor: dNLR＞3 and LDH>ULN;  3) Intermediate: either dNLR＞3 or LDH>ULN; |
| AAPR[3] | Albumin (g/L) / alkaline phosphatase (U/L) |
| SIRI[4] | absolute neutrophil count (10^9^/L) * absolute monocyte count (10^9^/L) / absolute lymphocyte count (10^9^/L) |
| SII[5] | Platelet count (10^9^/L) * absolute neutrophil count (10^9^/L) / absolute lymphocyte count (10^9^/L) |
| PNI[6] | Albumin (g/L) +5 * absolute lymphocyte count (10^9^/L) |
| PLR[1] | Platelet count (10^9^/L) / absolute lymphocyte count (10^9^/L) |

Abbreviations: Abbreviations: AAPR, albumin-to-alkaline phosphatase ratio; dNLR, derived neutrophil-to-lymphocyte ratio; LDH, lactate dehydrogenase; LIPI, lung immune prognostic index; NLR, neutrophil-to-lymphocyte ratio; PLR, platelet-to-lymphocyte ratio; SII, systemic immune-inflammation index; SIRI, systemic inflammation response index; ULN, upper limit of normal range.

Supplementary Table 2. Survival analysis for each systemic inflammation indexes.

|  | C-index | Corrected C-index | Coefficient | HR (95% CI) | p Value |
| --- | --- | --- | --- | --- | --- |
| dNLR (high vs. low) | 0.5666 | 0.5657 | 0.766 | 2.15 (1.84-2.51) | <0.001 |
| NLR (high vs. low) | 0.5737 | 0.5732 | 0.863 | 2.37 (2.03-2.77) | <0.001 |
| SIRI (high vs. low) | 0.5628 | 0.5619 | 0.851 | 2.34 (1.98-2.76) | <0.001 |
| SII (high vs. low) | 0.5951 | 0.5943 | 1.019 | 2.77 (2.37-3.24) | <0.001 |
| AAPR (high vs. low) | 0.5131 | 0.5104 | 0.158 | 1.17 (0.96-1.42) | 0.120 |
| PNI (high vs. low) | 0.5249 | 0.5260 | 0.263 | 1.30 (1.10-1.54) | 0.003 |
| PLR (high vs. low) | 0.5644 | 0.5654 | 0.732 | 2.08 (1.78-2.42) | <0.001 |
| LIPI (high vs. low) | 0.5316 | 0.5315 | 0.252 | 1.66 (1.34-2.04) | <0.001 |

Note: all comparisons were based on cutoff values derived from ROC analysis.

^*^ Statistical significance.

Abbreviations: AAPR, albumin-to-alkaline phosphatase ratio; dNLR, derived neutrophil-to-lymphocyte ratio; LIPI, lung immune prognostic index; NLR, neutrophil-to-lymphocyte ratio; PLR, platelet-to-lymphocyte ratio; SII, systemic immune-inflammation index; SIRI, systemic inflammation response index.

Supplementary Table 3. Characteristics of the development and validation populations.

| Variables | Total (n=2690) | Development population (n=1843) | Validation population (n=847) | P value |
| --- | --- | --- | --- | --- |
| Demographic | | | | |
| Age, Median (IQR) | 57 (48, 68) | 56 (47, 67) | 58 (49, 69) | 0.033^*^ |
| Sex, n (%) |  |  |  | 0.725 |
| Male | 1533 (57) | 1055 (57) | 478 (56) |  |
| Female | 1157 (43) | 788 (43) | 369 (44) |  |
| Procedural | | | | |
| Procedures, n (%) |  |  |  | < 0.001^*^ |
| First time AVF | 1800 (67) | 1270 (69) | 530 (63) |  |
| Repeat AVF | 398 (15) | 288 (16) | 110 (13) |  |
| PTA | 391 (15) | 209 (11) | 182 (21) |  |
| AV Graft | 101 (4) | 76 (4) | 25 (3) |  |
| Survival time (month), Median (IQR) | 18 (11, 28) | 24 (17, 31) | 12 (10, 15) | < 0.001^*^ |
| Clinical | | | | |
| Lym, Median (IQR) | 1 (0.75, 1.33) | 1.01 (0.76, 1.32) | 0.98 (0.75, 1.33) | 0.517 |
| PLT, Median (IQR) | 163 (120, 208) | 161 (117, 205) | 170 (124, 220) | 0.005 |
| Neu, Median (IQR) | 4.37 (3.38, 5.63) | 4.37 (3.37, 5.71) | 4.33 (3.41, 5.46) | 0.528 |
| Mon, Median (IQR) | 0.41 (0.3, 0.54) | 0.41 (0.3, 0.54) | 0.4 (0.3, 0.53) | 0.563 |
| WBC, Median (IQR) | 6.1 (4.97, 7.62) | 6.13 (4.97, 7.67) | 6.01 (4.96, 7.5) | 0.333 |
| HGB, Median (IQR) | 89 (75, 106) | 88 (74, 105) | 92 (76, 109.5) | < 0.001^*^ |
| ALB, Median (IQR) | 36.45 (31.3, 41.2) | 35.8 (31.1, 40.5) | 37.6 (32.2, 42.3) | < 0.001^*^ |
| ALP, Median (IQR) | 85 (67, 113) | 84 (66, 113) | 87 (68, 114) | 0.275 |
| LDH, Median (IQR) | 243 (195, 335) | 256 (202, 381) | 221 (186, 275) | < 0.001^*^ |
| CRP, Median (IQR) | 3.43 (1.11, 10.28) | 3.19 (1.08, 10.11) | 3.77 (1.21, 11.05) | 0.196 |
| Systemic immune-inflammation indicators | | | | |
| NLR, Median (IQR) | 4.24 (3.09, 6.17) | 4.21 (3.06, 6.22) | 4.36 (3.16, 6.03) | 0.788 |
| dNLR, Median (IQR) | 2.56 (1.96, 3.46) | 2.56 (1.92, 3.5) | 2.58 (2, 3.4) | 0.752 |
| AAPR, Median (IQR) | 0.42 (0.3, 0.55) | 0.41 (0.3, 0.55) | 0.42 (0.31, 0.55) | 0.332 |
| SIRI, Median (IQR) | 1.72 (1.09, 2.81) | 1.7 (1.08, 2.86) | 1.75 (1.11, 2.7) | 0.820 |
| SII, Median (IQR) | 688.96 (434.8, 1074.47) | 677.36 (428.81, 1075.4) | 726.14 (460.33, 1070.71) | 0.136 |
| PNI, Median (IQR) | 41.62 (36.54, 46.74) | 41.25 (36.21, 46.17) | 43 (37.45, 47.93) | < 0.001^*^ |
| PLR, Median (IQR) | 158.76 (115.56, 217.46) | 157.02 (113.78, 215.73) | 162.67 (119.89, 219.76) | 0.033 |
| LIPI, n (%) |  |  |  | < 0.001^*^ |
| Good | 965 (66) | 612 (33) | 353 (44) |  |
| Intermediate | 1183 (45) | 830 (45) | 353 (44) |  |
| Poor | 489 (34) | 390 (21) | 99 (12) |  |
| Survival status | | | | |
| Occluded | 305 (11) | 239 (13) | 66 (8) | < 0.001^*^ |
| Stenostic | 353 (13) | 261 (14) | 92 (11) |  |
| Normal | 2032 (75) | 1343 (73) | 689 (81) |  |

* Statistically significant.

Abbreviations: AAPR, albumin-to-alkaline phosphatase ratio; ALB, albumin; ALP, alkaline phosphatase; AV, arteriovenous; AVF, arteriovenous fistula; CRP, C reactive protein; dNLR, derived neutrophil-to-lymphocyte ratio; HGB, hemoglobulin; IQR, inter quartile range; LDH, lactate dehydrogenase; LIPI, lung immune prognostic index; Lym, lymphocyte; Mon, monocyte; n, number; Neu, neutrophil; NLR, neutrophil-to-lymphocyte ratio; PLR, platelet-to-lymphocyte ratio; PLT, platelet; PNI, prognostic nutritional index; PTA, percutaneous transluminal angioplasty; SII, systemic immune-inflammation index; SIRI, systemic inflammation response index.

Supplementary Figure 1. Distribution of systemic immune-inflammation indexes based on cut-off values determined by ROC analysis.


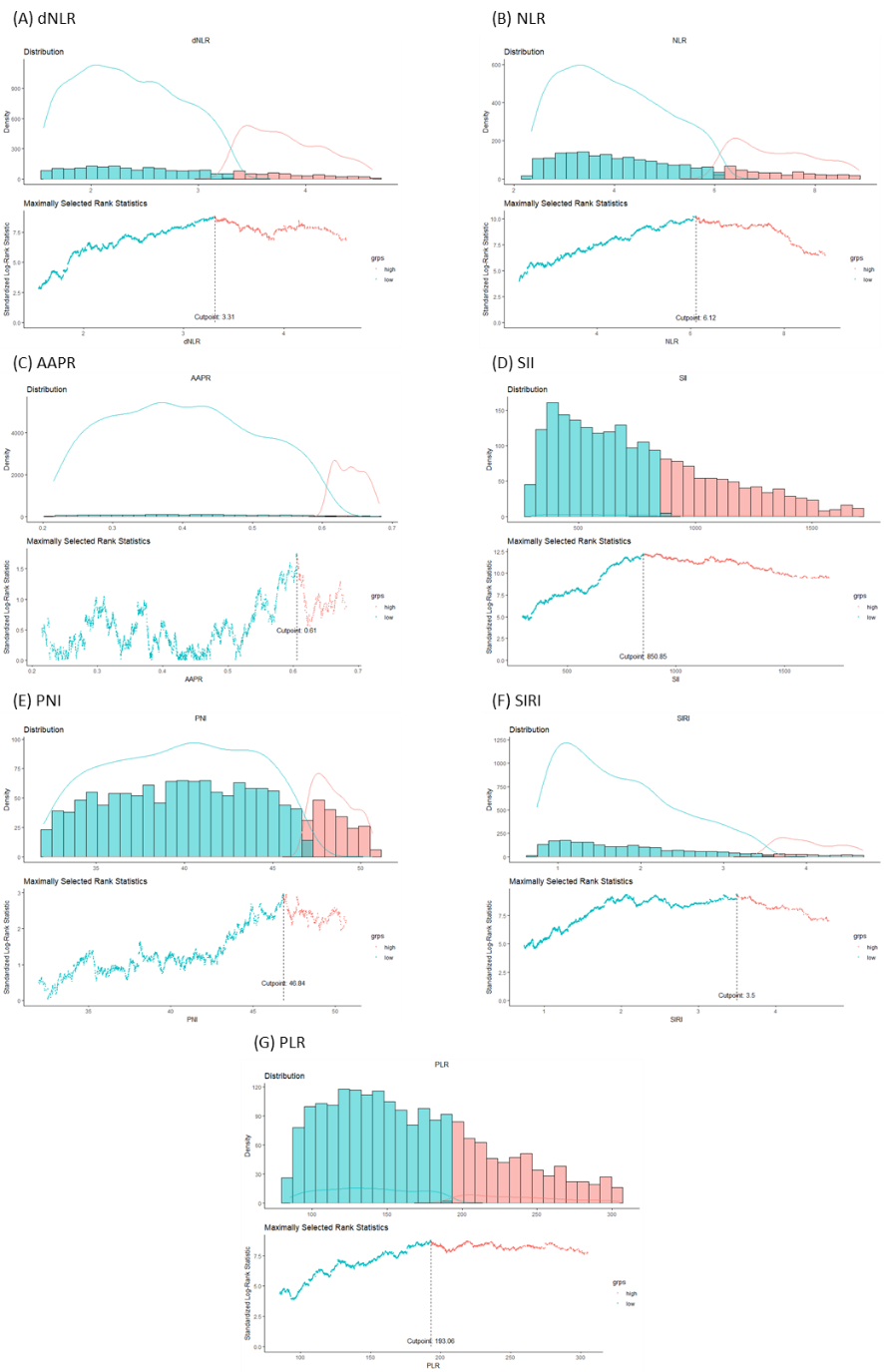


Supplementary Figure 2. ROC analysis for the final model to predict 6- and 12- month access survival in the development population.


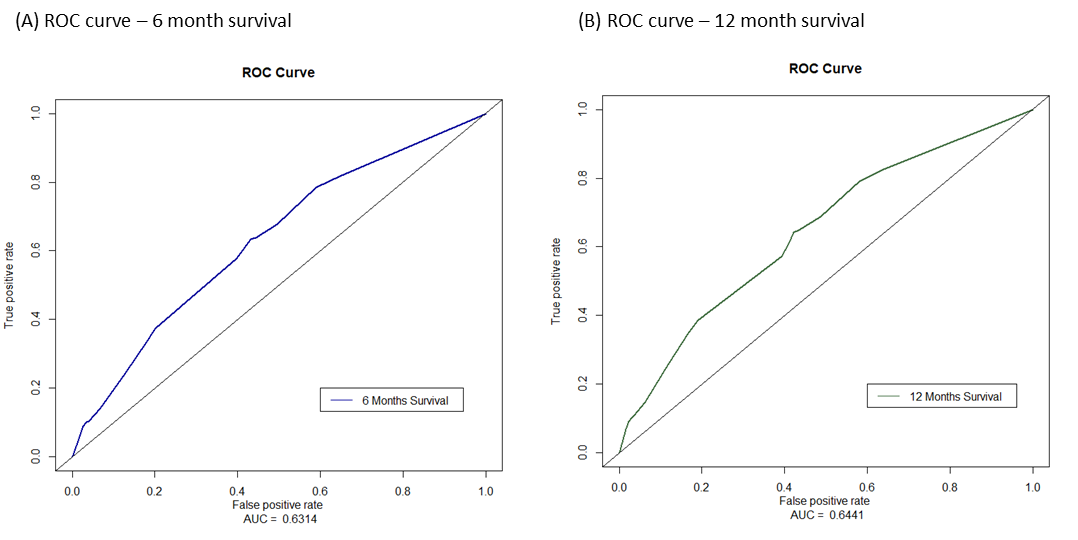


Note: The C-indexes of the final model to predict 6- and 12-months survival were 0.6314 (95% CI: 0.6249 – 0.6589) and 0.6441 (95% CI: 0.6212 – 0.6670), respectively.

Supplementary Figure 3. Calibration plots for the final model to predict 6- and 12- month access survival in the development population.


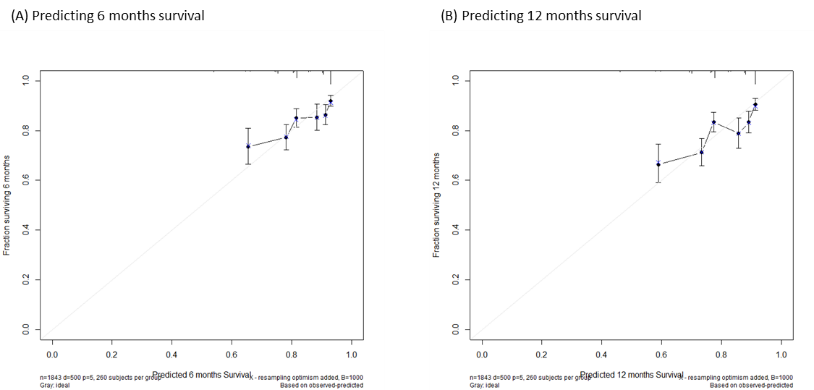


Supplementary Figure 4. DCA curves for the final model to predict 6- and 12- month access survival in the development population.


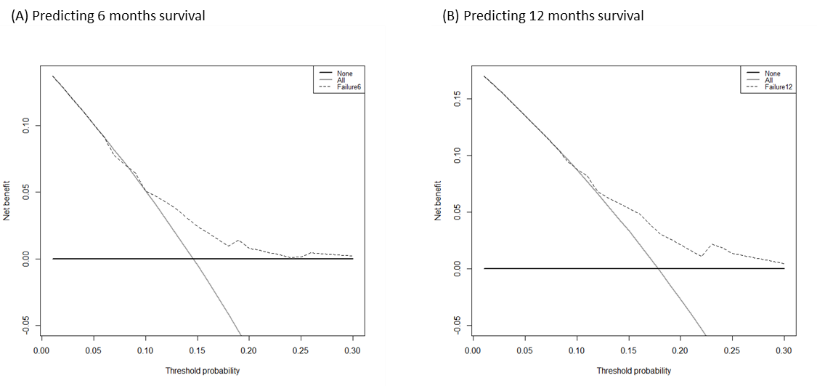


Note: The net benefits of this model to predict 6- and 12- access survival were 10% to 25% and 10% to 30%, respectively.

Supplementary Figure 5. Discrimination assessment of the prediction model in the external validation population.


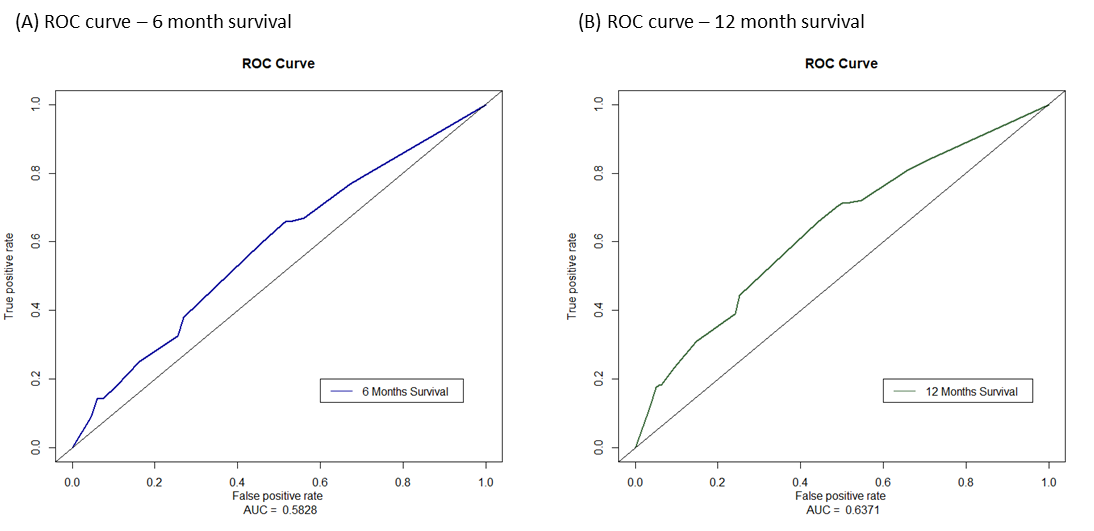


Note: The C-indexes of the final model to predict 6- and 12-months survival in the external validation were0.5828 and 0.6347, respectively.

Supplementary Figure 6. Calibration assessment of the prediction model in the external validation population.


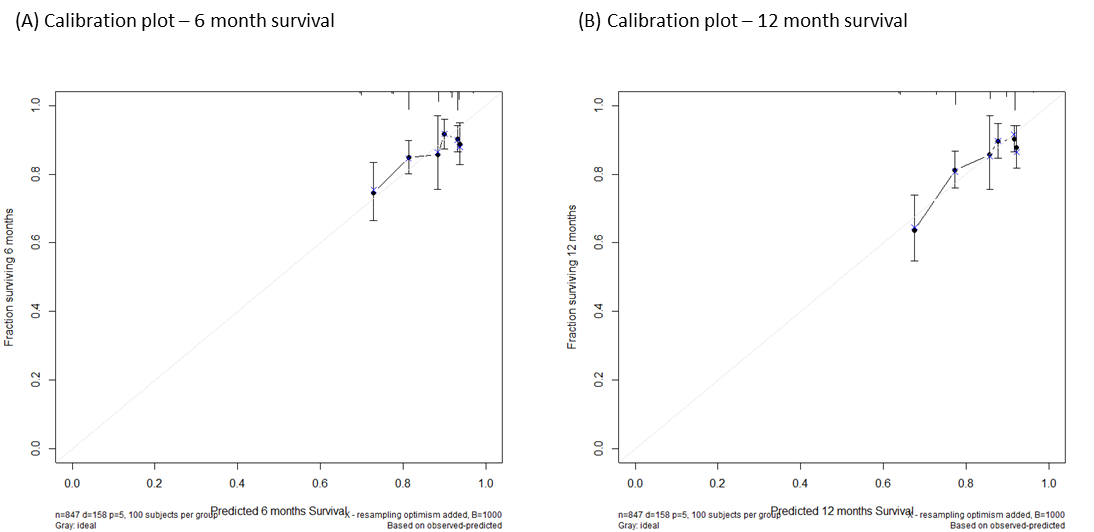


Supplementary Figure 7. DCA curves of the prediction model in the external validation population.


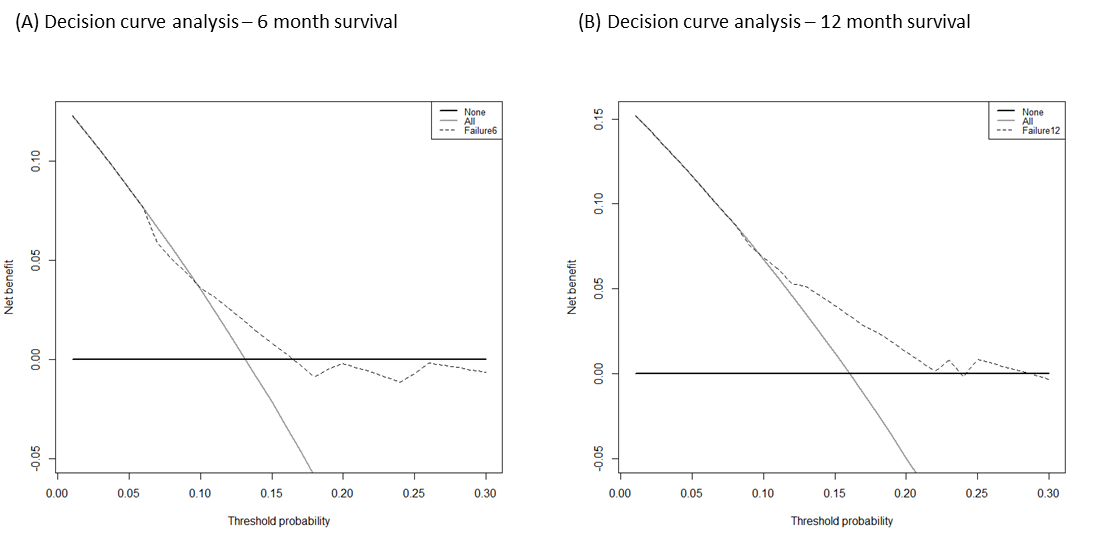


Note: The net benefits of this model to predict 6- and 12- access survival were 10% to 16% and 10% to 22%, respectively.

**References**

1. Ying, H.Q., et al., *The prognostic value of preoperative NLR, d-NLR, PLR and LMR for predicting clinical outcome in surgical colorectal cancer patients.* Med Oncol, 2014. **31**(12): p. 305.

2. Mezquita, L., et al., *Association of the Lung Immune Prognostic Index With Immune Checkpoint Inhibitor Outcomes in Patients With Advanced Non-Small Cell Lung Cancer.* JAMA Oncol, 2018. **4**(3): p. 351-357.

3. Zhang, X., et al., *Prognostic effect of albumin-to-alkaline phosphatase ratio on patients with hepatocellular carcinoma: a systematic review and meta-analysis.* Sci Rep, 2023. **13**(1): p. 1808.

4. Qi, Q., et al., *A novel systemic inflammation response index (SIRI) for predicting the survival of patients with pancreatic cancer after chemotherapy.* Cancer, 2016. **122**(14): p. 2158-67.

5. Hu, B., et al., *Systemic immune-inflammation index predicts prognosis of patients after curative resection for hepatocellular carcinoma.* Clin Cancer Res, 2014. **20**(23): p. 6212-22.

6. Zhao, P., et al., *Prognostic role of the prognostic nutritional index in patients with pancreatic cancer who underwent curative resection without preoperative neoadjuvant treatment: A systematic review and meta-analysis.* Front Surg, 2022. **9**: p. 992641.
